# Comparison of efficacy and safety of equivalent doses of remimazolam versus propofol for gastroscopy anesthesia in elderly patients

**DOI:** 10.1038/s41598-024-58294-2

**Published:** 2024-04-01

**Authors:** Di Chen, Min Liao, Xiao-ru Wu, Tang-yuan-meng Zhao, Hu Sun

**Affiliations:** 1 The Second Affiliated Hospital of Hainan Medical University, Haikou, China; 2 West China Hospital, Sichuan University, Chengdu, 610041 China

**Keywords:** Remimazolam, Elderly patients, Gastroscopy, Propofol, Effective dose, Drug discovery, Medical research

## Abstract

Remimazolam, a novel intravenous anesthetic, has been proven to be safe and efficacious in the gastroscopy setting among the elderly. However, reports comparing the effectiveness and safety of using equivalent doses of remimazolam with propofol have not been seen. The aim of this study was to compare the sedation efficacy and safety of the 95% effective doses (ED_95_) of remimazolam versus propofol combined with sufentanil in the gastroscopy setting among the elderly. In the first step of this two-step study, a modified up-and-down method was used to calculate the ED_95_ of remimazolam and propofol when combined with 0.1 µg/kg sufentanil in inhibiting body movement of elderly patients undergoing gastroscopy. In the second step, ED_95_ of both agents calculated in the first step were administered, endpoints of efficacy, safety, and incidence of adverse events were compared. A total of 46 individuals completed the first step. The ED_95_ of remimazolam was 0.163 mg/kg (95% CI 0.160–0.170 mg/kg), and that of propofol was 1.042 mg/kg (95% CI 1.007–1.112 mg/kg). In the second step, 240 patients completed the trial. The anesthetic effective rates of the remimazolam group and the propofol group were 78% and 83%, respectively, with no statistical difference (*P* = 0.312). Patients in the remimazolam group had more stable circulatory functions (*P* < 0.0001) and a lower incidence of pain on injection (3.3% vs. 19.5%, *P* < 0.0001). The incidence of hypotension was low in the remimazolam versus propofol group (15.6% vs. 39.0%, *P* < 0.0001). Overall adverse event was low in the remimazolam versus propofol group (21.3% vs. 62.7%, *P* < 0.0001).In this study, we found that when anesthesia was administered to elderly gastroscopy patients based on 95% effective doses of remimazolam and propofol, remimazolam was as effective as propofol, but was safer with a lower incidence of adverse events.

Study registration: Chinese Clinical Trial Registry, ChiCTR2000034234. Registered 29/06/2020, https://www.chictr.org.cn.

## Introduction

With the aging of population and the rapid development of digestive endoscopy technology, the proportion of elderly patients receiving painless gastroscopy has increased significantly^1^. However, general anesthetics may cause complications such as changes in systemic vasodilation, decreased peripheral circulatory resistance, myocardial depression, and varying degrees of respiratory depression among the elderly, increasing the risk of cardiovascular and cerebrovascular accidents and hypoxemia events^2,3^. Therefore, safe and effective anesthetic regimens are of great significance in the gastroscopy setting for this population.

Remimazolam is a new type of ultra-short-acting benzodiazepine, antagonizing gamma-aminobutyric acid type A (GABA_A_) receptors. It channels the influx of chloride ions, leading to neuron hyperpolarization, thereby inhibiting neuronal electrical activities^4^. When administered intravenously, it acts in the amygdala and reticular activating system, inhibiting polysynaptic pathways, ultimately curbing the central nervous system^5^. Preliminary studies have demonstrated that remimazolam has a rapid onset, a short duration of action, and little impact on the respiratory and circulatory systems. It is not metabolized by liver or kidney and is hydrolyzed by non-specific cholinesterase in plasma^6–8^. Thus, the agent could potentially be an ideal anesthetic for endoscopy.

Propofol is a classic intravenous anesthetic. Its advantages include fast onset of action, rapid recovery, and low incidence of postoperative nausea and vomiting. However, speedy intravenous infusion of propofol in large volume can easily lead to adverse events including varying degrees of respiratory and circulatory depression, muscle tremors, pain on injection, and low endoscopist satisfaction^9^, and the incidence of such events is higher in elderly patients^10,11^.

In recent years, some randomized, controlled, double-blind clinical trials have reported the safety and efficacy of remimazolam in elderly patients undergoing gastroscopy^12,13^. However, these studies are limited by high incidence of adverse events, insufficient rational for remimazolam dosage selection, inconsistent depth of sedation, and small sample sizes. Our team has completed several studies exploring this novel agent for painless gastroscopy^14–16^. Nevertheless, there is no prospective, randomized, controlled studies on the safety and efficacy of equivalent doses of remimazolam versus propofol combined with sufentanil in elderly patients receiving gastroscopy to date. This two-step study was designed based on our previous work. In the first step, we used a modified up-and-down strategy to estimate the ED_95_ equivalent doses of remimazolam and propofol for gastroscopy anesthesia in the elderly. In the second step, we conducted a randomized controlled trial comparing the safety and efficacy of equivalent doses of remimazolam versus propofol combined with sufentanil in this clinical setting, aiming to further optimize the anesthesia regimen for this population and to provide a reference for rational clinical use of drugs.

## Materials

This study was approved by the Ethics Committee of the Second Affiliated Hospital of Hainan Medical College (KY2020016) and registered in the China Clinical Trial Registry (ChiCTR: 2000034234). We obtained informed consent from all individual participants and their families. They each signed an informed consent form for anesthesia.

### Study population

Elderly inpatients and outpatients who underwent painless gastroscopy at the Second Affiliated Hospital of Hainan Medical College (a Grade 3A hospital) from October 2020 to November 2022 were enrolled.

### Inclusion criteria

(1) Aged 65–80 years, in either sex, with a body mass index (BMI) between 16 and 35 kg/m^2^; (2) able to undergo routine gastroscopy; (3) American Society of Anesthesiologist (ASA) grade of I-II before anesthesia, Mallampati class I or II; (4) no serious disease of the heart, lung, brain, liver (Normal liver function laboratory tests and imaging or in CHILD stage A), kidney (GFR of > 90) and other important organs, and no schizophrenia or severe depression.

### Exclusion criteria

(1) Allergic to propofol, sufentanil, benzodiazepines, or any component of study agents; (2) myasthenia gravis; (3) depressive state or schizophrenia; (4) severe respiratory diseases (such as acute chronic obstructive pulmonary disease, bronchial asthma attack, and obstructive sleep apnea hypopnea syndrome); (5) poorly controlled severe hypertension; (6) heart rate (HR) < 50 beats/min and atropine test positive or third-degree atrioventricular block; (7) sinus tachycardia at rest with HR > 120 beats/min or severe arrhythmia; (8) pyloric obstruction or high intestinal obstruction; (9) long history of alcohol or drug abuse; (10) contraindications for intravenous anesthesia.

### Rule-out criteria

(1) The duration of gastroscopy exceeding 30 min; (2) Perioperative serious adverse events or procedural accidents.

### Study protocol

In this two-step study, a randomized, double-blind, single-arm research was carried out in the first step. A random number table was used for participant enrollment, assigning to either the remimazolam effective dose group (RED group) or the propofol effective dose group (PED group). Sample sizes of these groups were determined when negative response and positive response alternated 7 times in the up-and-down experiment. The second step was a randomized controlled double-blind trial in which participants were assigned to either the remimazolam group (group R, n = 125) or the propofol group (group P, n = 125) per random number table.

### Anesthesia protocol and technical route

**First step**: Participants were enrolled in the order of computer-generated random numbers. All patients fasted for solids for 8 h and fasted for water for 2 h preoperatively. After patients entered the operating room, intravenous access was established, 0.9% sodium chloride injection 15 ml/(kg h) was administered intravenously for fluid maintenance with nasal cannula oxygenation (4 L/min). In a left decubitus position, parameters were measured (T_0_), including modified observer's assessment of alert/sedation (MOAA/S), mean arterial pressure (MAP), heart rate (HR), saturation of peripheral oxygen (SpO_2_), and respiratory rate (RR). Only 1 occurrence of hypotension was recorded for the same subject in the study regardless of the number of occurrences of hypotension, and the incidence of hypotension was equal to the number of occurrences of hypotension/total number of trials in the same group. In 60 s after oxygenation via nasal cannula, 0.1 µg/kg of sufentanil (*Sufentanil Citrate Injection, Rui Jing, Yichang Humanwell Pharmaceutical, Hubei, China; lot no. 31A050211*) was injected intravenously at a uniform rate , and after 180 s (T1) , preset doses of remimazolam (*Remimazolam Tosilate for Injection,Jiangsu Hengrui Pharmaceutical,Jiangsu, China;lot no. 200202AK*) or propofol (*Propofol Injectable Emulsion,Guo Rui Pharmaceutical,Si Chuan, China; lot no.2211281*) were administered intravenously with an injection duration of 60 s. 120 s later(T_2_^*^), gastroscopy was performed when the eyelash response disappeared and the MOAA/S ≤ 1 (T_3_^#^). During anesthesia we always checked the patient 60 s until full recovery.

Following the modified up-and-down method, doses of intravenous anesthetics were determined for each patient. According to preliminary results, the initial doses for the RED group and the PED group were 0.15 mg/kg and 0.8 mg/kg with an increment of 0.01 mg/kg and 0.1 mg/kg, respectively. When a patient's body movement response was negative for gastroscope insertion, anesthesia was deemed effective and a de-escalation of one increment unit would be given to the next patient; otherwise, in case of ineffective anesthesia, an escalation of one increment unit would be administered. The study was terminated when negative response and positive response alternated 7 times. The definition of a positive response, or effective anesthesia, referred to reactions such as coughing, nausea, vomiting, and/or body movement occurred during or within 120 s after gastroscope insertion. The effective dose is defined as the dose administered when the patient is anesthetized effectively with either remimazolam or propofol.

Endoscopic exams were completed by attending physicians specializing in gastrointestinal endoscopy who have worked for at least three years. Anesthesia was performed by two anesthesiologists, one of whom was responsible for administering anesthetics and handling potential adverse events, and the other for recording pre-specified indicators.

*T_2_ was defined as the time when there was no gastroscope placement at 120 s after the completion of the experimental drug injection.

^#^T3 is defined as the time at which the gastroscope is placed, when the subject's vital signs change accordingly because the gastroscope enters the subject's laryngopharynx.

**Second step**: In this randomized controlled double-blind trial, based on preliminary results, a total of 250 participants were enrolled in this step after sample size calculation, and were randomly assigned to either group R (n = 125) or group P (n = 125). For randomization we used EXCEL software. Subjects were numbered from 1 to 250 according to the serial number of the patients included in the study, and then the RANDBETWEEN function was used to randomly generate a number between 1 and 250 corresponding to the serial number, if the number generated corresponding to the serial number was odd it was categorized as the remimazolam group, if it was even it was categorized as the propofol group.

Blinding we used a double-blind method, that is, the subject patients did not know which group they were assigned to, while the trial implementer was done by 2 anesthesiologists, one anesthesiologist was responsible for administering anesthesia medication and anesthesia management, and the other anesthesiologist was responsible for recording the vital signs and adverse reactions. The definition of observables during anesthesia and anesthesia effectiveness was identical to that of the first phase.

Pre-anesthesia preparation and monitoring were the same as in the first step. In 60 s after oxygenation via nasal cannula, 0.1 µg/kg of sufentanil was injected intravenously, and after 180 s, 0.17 mg/kg of remimazolam and 1.112 mg/kg of propofol were administered to group R and group P, respectively. Doses of the two groups were determined based on the highest value within the 95% confidence interval of each anesthetic’s ED_95_ calculated in the first step and preliminary results.

### Remedial measures and discharge criteria

Remedial measures: When the anesthesia failed, added 2.5 mg/time as needed for the group R, and 1/2 of the initial dose for the group P. Sedation efficacy was assessed every 60 s until satisfaction.

Discharge criteria: The postanesthesia discharge scoring system (PADS) was used to evaluate whether the patient met the criteria for discharge^17^.

### Management of adverse events during anesthesia

When a patient's systolic blood pressure (SBP) was > 180 mmHg during anesthesia, urapidil 5 mg/time was injected intravenously. When SBP dropped below 90 mmHg during anesthesia, ephedrine 6 mg was administered intravenously. In case of HR < 50 beats/min, atropine 0.5 mg was given intravenously. When SpO_2_ decreased < 90%, the oxygen flow was elevated. Mask ventilation or jaw lift maneuver would be performed to open the airway. If necessary, tracheal intubation was carried out to assist breathing, ensuring that oxygen saturation remained within the normal range. When a patient developed perioperative nausea and vomiting, ondansetron 4 mg was administered intravenously.

### Outcomes

**Primary outcomes**: Safety indicators (incidence of adverse events) during anesthesia in elderly patients. MOAA/S, MAP, HR, SpO_2_ and RR at T_0_ (patients entering the endoscopy room), T_1_ (3 min after sufentanil injection), T_2_ (2 min after remimazolam or propofol injection), T_3_ (gastroscope inserting into the pharyngeal cavity), T_4_ (immediately after gastroscope withdrawal) and T_5_ (patients fully woke up). In addition, we recorded other adverse events including hypotension (30% or more drop in blood pressure from baseline), respiratory depression (RR 8 times/min or SpO_2_ < 90%), nausea and vomiting, pain on intravenous injection (dodging of the ipsilateral upper limb or pain at the injection site reported by patients after administration), intraoperative awareness, and postoperative delirium and patients' recovery time during anesthesia (the time from the last intravenous injection of anesthetic to three consecutive MOAA/S = 5 evaluations).

**Secondary outcome**: Anesthetic effective rates of both groups.

### Statistical analysis

Statistical analysis was performed using SPSS Statistics 25™ (SPSS Inc., Chicago, IL, USA). Normally distributed continuous variables were expressed as means ± standard deviations (SD, $\overline{x} \pm s$) and were compared by Independent Samples *t* test. Non-normally distributed data were presented as medians (interquartile ranges, IQR) and were analyzed by Mann–Whitney U test. Categorical variables were expressed as frequencies (%) and were compared using Fischer’s exact test or chi-square test. ED_50_ and ED_95_ for the two groups were calculated using the probit method. Repeated measures ANOVA was used to analyze hemodynamic and respiratory changes. Two-way ANOVA was used to analyze hemodynamic and respiratory changes. If the effect was considered significant, Bonferroni post-tests was performed to compare replicate means by row.

PASS 15.0.5 (NCSS, LLC, Kaysville, USA) was used for sample size calculations. Since the definition of adverse events is not entirely consistent across studies, and for painless gastroscopy anesthesiologists are mainly concerned with their respiratory-circulatory adverse effects (hypotension being the most common)^7,18,19^. Therefore, this study focused on hypotension. According to the results of the phase I trial and pretest, combined with relevant literature reports, the hypotension rate in the remimazolam group was 13.04%, and the incidence of hypotension in the propofol group was 42.86%, with a set test level of α = 0.05 (bilateral test), a type II error of β = 0.9, and a degree of certainty (test efficacy) of 1 − β = 90%, and a sample rejection rate of 10% for each group. 114 subjects needed to be included, and we did the calculations based on the incidence of hypotension based on the results of the phase I trial and the phase II pretest, combined with reference #12 and the incidence reported in several papers, and we chose the value with the smallest incidence for our calculations.

Figures were generated using GraphPad Prism 9.5.1 version (GraphPad Software LLC, San Diego, CA, USA). A *P* value < 0.05 is statistically significant.

### Ethics approval and consent to participate

This study was registered at http://www.chictr.org.cn (ChiCTR 2000034234). The study protocol followed relevant guidelines. The trail was conducted in accordance with the principles of the Institutional Research Board of the authorized hospital. Written informed consent was obtained from all patients.

## Results

### Patient information

**Figure 1:**
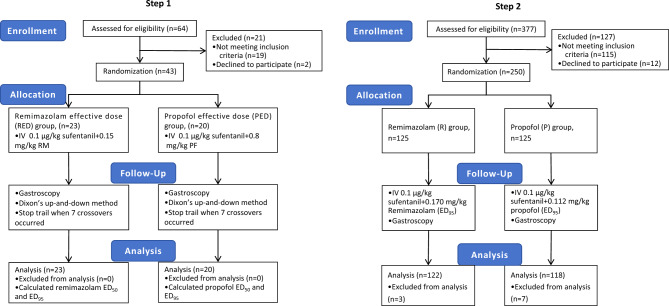
Flow chart of patient enrollment, allocation, and analysis. Step 1: Flowchart of patient enrollment, allocation, follow-up and analysis to calculate remimazolam and propofol ED_95_ by Dixon’s up-and-down method. Step 2: Flowchart of patient enrollment, allocation, follow-up and analysis to compare the efficacy and safety of remimazolam and propofol for anesthesia in elderly patients undergoing gastroscopy using the dosage of ED_95_ of remimazolam and propofol, respectively. IV, intravenous; RM, remimazolam; PF, propofol.

All recruited patients completed the trial, as shown in Fig. 1.

#### First step

Twenty-three patients in the RED group and 20 in the PED group completed the trial. No statistically significant differences were found for sex, age, weight, height, or BMI between the two groups, as shown in Table 1.

**Table 1:** Basic characteristics of patients in the two groups in step 1.

Variables	Group RED (n = 23)	Group PED (n = 20)	*P* value
Sex (M/F)	13/10	13/7	0.571
Age (years)	72.2 ± 4.6	72.7 ± 4.9	0.772
Height (cm)	163.6 ± 12.3	165.7 ± 11.1	0.479
Weight (kg)	59.9 ± 7.1	59.6 ± 9.8	0.898
BMI (kg/m^2^)	23.4 ± 2.7	22.6 ± 2.8	0.372
ASA grade			0.935
I	6 (26.1%)	5 (25%)	
II	17 (73.9%)	15 (75%)	

Values were expressed as the $\overline{x} \pm s$, median (IQR), or the number of patients and percentage. M, male; F, female; BMI, body mass index; ASA, American Society of Anesthesiologists. No significant differences were found for these variables between the two groups.

#### Second step

In total, 250 patients were included in this step. In group R, 122 patients completed the study, and 3 were ruled out due to long gastroscopy duration. In group P, 118 patients completed, and 7 were ruled out (5 due to severe hypotension and 2 due to long gastroscopy duration). No statistically significant differences were found for sex, age, weight, height, or BMI between the two groups, as shown in Table 2. Gastroscopy time ranged from 3.1 to 10.6 (4.7 ± 1.8) min in the remimazolam group and from 2.7 to 10.1 (4.5 ± 1.7) min in the propofol group, and there was no statistically significant difference in comparison between the groups (*P* = 0.6927).

**Table 2:** Basic characteristics of patients in the two groups in step 2.

Variables	Group RED (n = 122)	Group PED (n = 118)	*P* value
Sex (M/F)	66/56	62/56	0.809
Age (Years)	71.9 ± 5.0	71.7 ± 5.1	0.757
Height (cm)	163.6 ± 12.3	165.7 ± 11.1	0.98
Weight (kg)	57.8 ± 10.1	58.5 ± 9.6	0.621
BMI (kg/m^2^)	22.2 ± 3.0	22.5 ± 2.9	0.98
ASA grade			0.887
I	32 (26.2%)	30 (25.4%)	
II	90 (73.8%)	88 (74.6%)	

Values were expressed as the, median (IQR), or the number of patients and percentage. M, male; F, female; BMI, body mass index; ASA, American Society of Anesthesiologists. No significant differences were found for these variables between the two groups.

### Effective doses and effective rates

#### Effective doses

**Figure 2:**
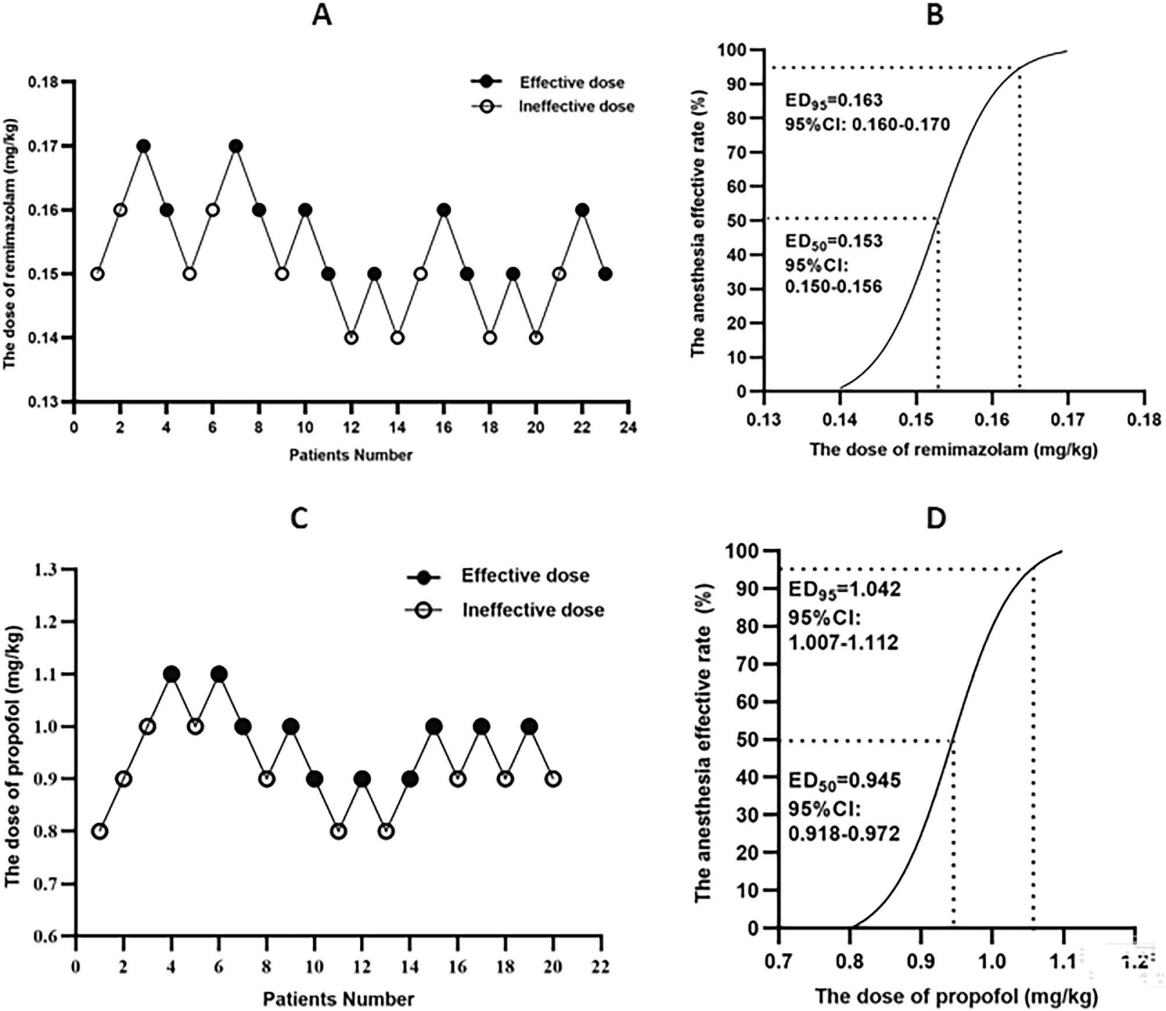
Sequential dose adjustment of remimazolam and propofol by Dixon’s up-and-down method and their corresponding dose–effect curves. A: Dixon's up-and-down method for remimazolam; Effective dose (mark●); Ineffective dose (mark○). B: Dose–effect curve of remimazolam. C: Dixon's up-and-down method for propofol; Effective dose (mark●); Ineffective dose (mark○). D: Dose–effect curve of propofol.

Among the 23 patients in the RED group, 11 were ineffective under anesthesia and 12 were effective. The up-and-down dose-finding process of remimazolam combined with 0.1 µg/kg sufentanil for elderly patients undergoing gastroscopy is shown in Fig. 2 A, and the corresponding dose–effect fitting curve is shown in Fig. 2B. The ED_50_ and ED_95_ of remimazolam combined with 0.1 µg/kg sufentanil in inhibiting body movement responses in this clinical scenario were 0.153 mg/kg (95% CI 0.150–0.156 mg/kg) and 0.163 mg/kg (95% CI 0.160–0.170 mg/kg), respectively.

Among the 20 patients in the PED group, 11 were ineffective under anesthesia and 9 were effective. The up-and-down dose-finding process of propofol combined with 0.1 µg/kg sufentanil for elderly patients undergoing gastroscopy is shown in Fig. 2C, and the corresponding dose–effect fitting curve is shown in Fig. 2D. The ED_50_ and ED_95_ of propofol combined with 0.1 µg/kg sufentanil in inhibiting body movement responses in this clinical scenario were 0.945 mg/kg (95% CI 0.918–0.972 mg/kg) and 1.042 mg/kg (95% CI 1.007–1.112 mg/kg), respectively.

### Comparison of effective rates between group R and group P

A total of 240 patients completed the second step. The effective anesthetic rate of group R was 78%, and 83% in group P. There was no statistically significant difference between the two groups (*P* = 0.312).

### Comparison of safety indicators between group R and group P

**Figure 3:**
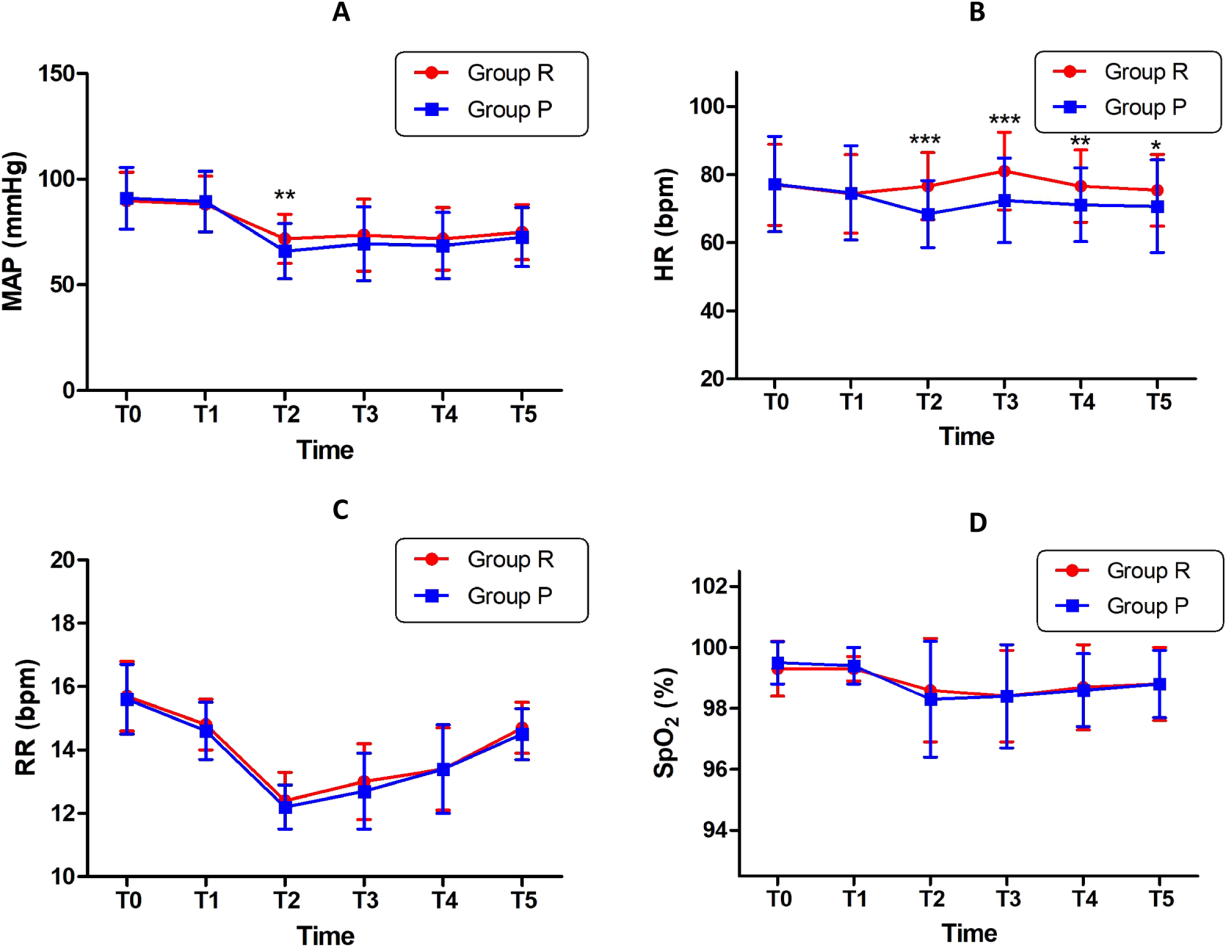
Comparison of MAP, HR, RR and SpO_2_ between the two groups at different time points.

By comparing MAP, HR, RR, and SpO_2_ at the same time points between the two groups, we found that the decrease in MAP in group R was smaller than that in group P (*P* < 0.0001). HR changes in group R showed an upward trend, while those in group P showed a downward trend. Differences between the two groups were statistically significant (*P* < 0.0001). In terms of RR and SpO_2_, both group R and group P showed a decreasing trend, and there was no statistically significant difference between the two groups (*P* = 0.639, *P* = 0.124 respectively). The RR value decreased most obviously at T_2_ (*P* < 0.0001). See Fig. 3. A: MAP of time effect was statistically significant (*P* < 0.0001), in T2 MAP of drug effect was significant (*P* < 0.01); B: HR of time effect was statistically significant (*P* < 0.0001), HR of drug effect was significant in T2,T3 (*P* < 0.001), T4 (*P* < 0.01) and T5 (*P* < 0.05); C: RR of time effect was statistically significant (*P* < 0.0001), RR of drug effect was not significant (*P* > 0.05); D: SpO_2_ of time effect was statistically significant (*P* < 0.001), SpO_2_ of drug effect was not significant (*P* > 0.05).

The recovery time of group R was (10.7 ± 3.8) min, significantly longer than that of group P (9.2 ± 3.3 min), *P* = 0.002.

### Comparison of adverse events between group R and group P

The overall adverse event rate was 21.3 versus 62.7% in the remimazolam and propofol groups, respectively, *P* < 0.0001.The incidence rates of hypotension and pain on injection in group R were significantly lower than those in group P (*P* < 0.0001). Among the adverse events observed, no symptoms such as nausea and vomiting (PONV), respiratory depression, intraoperative awareness, and postoperative delirium (POD) were found, as shown in Table 3.

**Table 3:** Comparison of adverse events between the two groups.

Adverse events	Group R (n = 122)	Group P (n = 118)	*P* value
Hypotension	19 (15.6%)	46 (39.0%)	< 0.0001
Pain on injection	4 (3.3%)	23 (19.5%)	< 0.0001
PONV	3 (2.5%)	5 (4.2%)	0.443
Respiratory depression	0	0	
Intraoperative awareness	0	0	
POD	0	0	

Values were expressed as the number of patients and percentage. No significant differences except Hypotension and Pain on injection were found in these variables between the two groups.

## Discussion

Advanced age significantly increases perioperative morbidity and mortality in elderly patients^20^ and has been confirmed to be an independent risk factor for anesthesia-related adverse events^21,22^. Studies have shown that compared with propofol, remimazolam is more effective in sedation and impacts less on the respiratory and circulatory systems^23^, and its advantages may be more prominent when used for anesthesia and sedation in the elderly^13^. However, previous randomized controlled studies did not compare remimazolam and propofol at equivalent doses, rendering results not ideal. Therefore, we designed a randomized, controlled, double-blind trial to compare 0.1 µg/kg sufentanil combined with equivalent doses of remimazolam or propofol for elderly patients undergoing gastroscopy to validate the efficacy and safety of both regimens in this clinical scenario.

A modified up-and-down dose-finding method was applied to calculate the effective doses of remimazolam and propofol combined with 0.1 µg/kg sufentanil in inhibiting body movement responses in elderly patients undergoing painless gastroscopy. With this method, precise optimal effective doses could be calculated with a smaller sample size while ensuring the depth of anesthesia, maximizing patient safety. Results showed that the ED_95_ of 0.1 µg/kg sufentanil combined with remimazolam or propofol to inhibit body movement responses of elderly patients undergoing painless gastroscopy were 0.163 mg/kg (95% CI 0.160–0.170 mg/kg) and 1.042 mg/kg (95% CI 1.007–1.112 mg/kg), respectively, lower than those reported by Enci et al.^12^. The analgesic used in this study was low-dose remifentanil (0.2 µg/kg), so the doses of remimazolam and propofol required were relatively high. The effective dose of remimazolam in our study was significantly lower and more precise than those reported by Cao et al.^24^, Borkett et al.^25^, Dai et al.^26^, Tan et al.^27^ and Zhang et al.^28^. Since research methods and participants of these studies varied, these results were not comparable.

In the second step, we used the upper limit of the 95% confidence interval for the equivalent doses of remimazolam (0.170 mg/kg) and propofol (1.112 mg/kg) found in the first step as the induction dose of intravenous anesthesia. After validation with a larger sample size in the second step, our results showed that the effective rate of remimazolam was 78% and that of propofol was 83%, both of which were lower than the 95% standard that should be theoretically achieved. This suggests that the 95% effective dose calculated using the probit regression model for real-world clinical practices with larger samples may be lower than the theoretical value. Our results were similar to other studies such as Hu et al.^13^. In addition, although the effective rates did not reach the theoretical target using these induction doses, there was no statistical difference between the two groups, maintaining equivalent doses during induction. Therefore, to pursue higher anesthesia efficacy, we need to appropriately increase the dose of intravenous anesthetics in clinical practices.

Perioperative hypotension and bradycardia are the most common sedation-related complications during endoscopy^29^. This study showed that compared with the propofol group, an equivalent dose of remimazolam yielded stabler MAP values, significantly higher HR at the same time points, and smoother circulatory functions. This is consistent with previous research^7,26,27,30^. Although MAP and HR in both groups changed from baseline after anesthesia, this advantage of remimazolam at equivalent doses can effectively reduce the possibility of organ damage in elderly patients caused by circulatory suppression, reducing the occurrence of related systemic complications. The reason for such changes may be that remimazolam maintains the balance of sympathetic and parasympathetic activities and has a slight effect on cardiac repolarization and myocardial contraction^6,31^.

On the other hand, a meta-analysis concluded that when the dose of remimazolam was 0.15–0.4 mg/kg, the incidence of respiratory depression was lower than that of propofol^7,28^. However, our study found that although the RR and SpO_2_ of both groups decreased after anesthesia, and the reduction in the remimazolam group was smaller than that of the propofol group at most time points, there was no statistical between-group difference at any time point. This may be due to the fact that a larger dose of sufentanil was used in this study, equivalent doses of both sedatives were lower, and the comparative advantage of remimazolam in reducing respiratory depression over propofol was not prominent. The dose of propofol was 1.112 mg/kg, much lower than that used in previous studies^7,28^. It has been concluded that a rapid administration of high-dose propofol may cause significant respiratory depression^31^, rendering a higher rate of this adverse event in previous studies using propofol.

In our study, the incidences of hypotension (15.6% vs. 39%) and pain on injection (3.3% vs. 19.5%) were significantly lower in the remimazolam group than in the propofol group. This result is consistent with Shi et al.^32^ and Xiao et al.^33^, showing that the use of remimazolam may significantly increase circulatory stability and avoid suboptimal patient experience during anesthesia by reducing the incidence of pain on intravenous injection. No adverse events such as nausea and vomiting, hypoxemia, intraoperative awareness, and postoperative delirium occurred in either group. However, some studies have found that as the single intravenous dose of remimazolam increases, the incidence of drug-related adverse events, including hypotension and respiratory depression, also increases^26^. Thus, in clinical practices, we need to be alerted that excessive doses of remimazolam may lead to significant suppression of respiratory and circulatory systems, and vital sign monitoring should be strengthened in this setting.

We found that the recovery time in the remimazolam group was (10.7 ± 3.8) min and that in the propofol group was (9.2 ± 3.3) min. The average recovery time of the remimazolam group was 90 s longer than the other group, but this difference was of no clinical significance. Our results are consistent with those reported by Doi et al.^33^, Liu et al.^34^, and Zhang et al.^30^. Pharmacokinetically, remimazolam is hydrolyzed by non-specific cholinesterase in plasma and is not metabolized in liver or kidney. Therefore, it is superior to propofol when used in patients with liver and kidney dysfunction. As a benzodiazepine sedative, the sedative effects of remimazolam can be quickly and completely reversed by flumazenil, with short and completely controllable recovery times^7,35^. Thus, the combination of the two agents may be an effective regimen to precisely control the level of anesthesia.

There are four limitations of this study. (1) The sample size of the first step is small, and a larger-scale study is needed to confirm the effective doses of remimazolam and propofol. (2) This study did not include elderly patients with ASA grade III or above. (3) This study did not use flumazenil to antagonize remimazolam, not reflecting the pharmacological advantage of remimazolam’s controllable sedation. (4) The results of the study have some limitations due to racial differences and exclusion criteria.

## Conclusion

In summary, the ED95 of 0.1 µg/kg sufentanil combined with remimazolam or propofol in inhibiting responses during gastroscopy among the elderly were 0.163 mg/kg (95% CI 0.160–0.170 mg/kg) and 1.042 mg/kg (95% CI 1.007–1.112 mg/kg), respectively. Moreover, when 0.170 mg/kg remimazolam and 1.112 mg/kg propofol were administered, there was no significant difference in the anesthetic effective rate between the two groups in inhibiting body movement response during gastroscopy in elderly patients. However, remimazolam enabled stabler circulatory function and fewer adverse events. Therefore, it is more beneficial than propofol when applied in gastroscopy among the elderly.

## Data Availability

The datasets used and/or analysed during the current study available from the corresponding author on reasonable request.
